# Motion capture data of six jump-landings, fatigued and non-fatigued, after anterior cruciate ligament injury

**DOI:** 10.1038/s41597-025-05934-5

**Published:** 2025-10-15

**Authors:** Maité Calisti, Maurice Mohr, Peter Federolf

**Affiliations:** Department of Sport Science, University of Innsbruck, Innsbruck, Austria

**Keywords:** Biophysics, Risk factors

## Abstract

Anterior cruciate ligament (ACL) injuries are common, and re-injuries remain high despite advances in rehabilitation. Return-to-sport (RTS) assessments focus on strength, clinical and hop tests, and time-based criteria but often exclude objective movement quality measures. Biomechanical deficits during jump-landings can persist post-reconstruction, contributing to re-injury risk. Fatigue further alters neuromuscular control, potentially exacerbating risk-related movement patterns, yet most RTS tests are conducted in non-fatigued states. This study introduces a motion dataset of 2199 trials across six bilateral (countermovement jump, drop jump) and unilateral (forward hop, countermovement jump, cross-over hop, 90° medial rotation hop) jump-landing tasks, performed under fatigued and non-fatigued conditions. The dataset includes 3D motion capture and ground reaction force data, including full-body inverse kinematics data (joint angles: knee, hip flexion, abduction, rotation, ankle flexion, trunk and pelvis) processed in OpenSim software for 43 participants comprising individuals with prior ACL injury (n = 21) and healthy controls (n = 22). The dataset enables detailed analyses of jump-landing biomechanics under fatigue, aiming to improve RTS decision-making to reduce re-injury risk.

## Background & Summary

An anterior cruciate ligament (ACL) injury is one of the most common knee injuries worldwide^1^. Despite advancements in prevention and rehabilitation, initial and re-injury rates remain high^2^. The return-to-sport (RTS) clearance continuum following ACL injuries involves a multifaceted evaluation process at the final stage of rehabilitation to determine if an individual can safely resume physical activity or competition^3–5^. Common RTS criteria include time since injury, strength testing, and clinical examinations^3,6,7^. While additional tests, such as hop performance and movement quality analysis, have been explored in ACL injury research and rehabilitation^8,9^, they are not consistently integrated in the RTS decision-making in a clinical setting^3^. Emerging evidence suggests that biomechanical deficits persist for years after ACL reconstruction (ACLR), causing asymmetries in landing mechanics and compensatory strategies, which both may contribute to an increased risk of re-injury^10,11^.

Additionally, fatigue plays a critical role in the biomechanics of human movement and RTS outcomes. Fatigue-induced neuromuscular changes, such as reduced knee flexion angles, hip flexion angles, and increased valgus, increase re-injury risk^12,13^. Yet, RTS criteria are typically evaluated under non-fatigued conditions^14,15^, which may fail to predict performance under stress.

Despite meeting RTS criteria, individuals remain at increased risk of sustaining a second ACL injury^16^. Several factors might contribute to this elevated risk. First, there is no consensus on which specific movement-related biomechanical variables most accurately quantify movement quality or reliably distinguish between “good” and “poor” movement patterns after ACL injury. Parameters such as knee valgus angle, flexion angle, and symmetry indices have all been proposed, but clear thresholds in the RTS testing are lacking, limiting their application in clinical settings.

Another issue is the selection of a sensitive jump-landing task for evaluating movement quality, particularly in clinical environments where space and time may be limited. While studies have examined the sensitivity of various jump tests in terms of performance and asymmetries^17,18^, a clear recommendation is lacking. Commonly used tasks such as the single-leg hop, drop jump, and vertical jump are all employed, and while some studies have highlighted specific tasks as more sensitive in detecting movement deficits, no clear consensus has been reached. For instance, Zarro *et al*. found that the single-leg vertical hop for height revealed significantly greater limb asymmetries than horizontal hop tests in athletes after ACL reconstruction^19^. Similarly, Dingenen *et al*. reported that medial and rotational hop tests were more likely to expose limb asymmetries in ACL-reconstructed athletes compared to forward-directed hop tests^20^.

Furthermore, the influence of fatigue on both movement quality and the validity of RTS criteria remains a subject of debate. Some evidence suggests that fatigue exacerbates movement deficits, decreases knee stability, and increases tibial translation, whereas other studies report inconsistent effects^21–23^. This inconsistency makes it difficult to standardize fatigue protocols or interpret their clinical relevance for RTS decision-making.

To address some of the issues described above and to support an evidence-based RTS assessment post ACL-injury, this report describes a comprehensive shared dataset comprising movement data from six commonly used bilateral or unilateral jump-landing tasks performed under both fatigued and non-fatigued conditions. The data include motion capture and ground reaction force data from 43 participants, categorized into two groups: individuals with a history of ACL injury and healthy controls. This dataset is intended to facilitate the analysis of movement quality during various jump-landing tasks, particularly regarding the impact of fatigue on biomechanics. Such analyses aim to deepen the understanding of whole-body movement quality and the role of fatigue in RTS assessments.

While several datasets address lower-limb biomechanics, none, to our knowledge, combine multiple jump-landing tasks with both fatigued and non-fatigued conditions in ACL-injured individuals. Existing datasets focus on gait or running^24,25^, general screening such as stability measures using drop jump, and hop downs, without fatigue^26^, or rehabilitation exercises for patients with knee pathologies using wearable sensors^27^. Others include fatigue on walking in healthy individuals^28^. Another study from Wang and colleagues examined single-leg landings and vertical jumps in uninjured individuals using IMUs, but without fatigue or ACL-specific focus^29^.

In a first analysis of this dataset^30^ our research group has addressed the issue regarding the selection and sensitivity of jump-landing tasks for identifying deficits in movement quality. In this analysis, it was investigated which jump-landing task and which fatigue status can predict most accurately whether jump-landing kinematics belong to an individual with a previous ACL injury vs. to healthy participants. The findings indicate that fatigue generally led to improved prediction accuracy, with the most accurate results obtained for the single-leg hop performed in a fatigued state.

However, other issues for RTS decision-making described above, e.g. the selection of the most appropriate biomechanical variables to assess movement quality could not yet be addressed. The goal of this manuscript is therefore to describe the full dataset in detail, including participant characteristics, the laboratory setup, data collection protocols, pre-processing procedures, and file structure, to facilitate its broader use in the clinical biomechanics community. The present work serves as a technical data descriptor, providing the full dataset and enabling a broader range of future analyses beyond joint kinematics, such as ground reaction force–based metrics, symmetry assessments, or investigations into the role of leg dominance and the contralateral limb.

## Methods

### Participants

A total 44 participants were initially included in this study. This convenience sample consisted of 22 individuals with a history of ACL injury (ACL group: 11 males and 11 females; age range: 20–36), and 22 control participants without previous ACL injuries (control group: 10 males and 12 females; age range: 19–31). The study was conducted between June 2022 and February 2023. One participant (sub5, ACL group, male) was excluded due to excessive marker movement caused by sweating, resulting in a final sample of 43 participants.

All ACL injuries involved complete ruptures. 12 participants reported that their injury occurred while skiing, two during football, and one each during handball, beach volleyball, wrestling, jumping, bouldering, trampolining, and hurdling. The specific injury mechanism (e.g., contact vs. non-contact) was not explicitly collected. 19 ACL participants had undergone surgical reconstruction (ACLR), while two were treated conservatively without surgery (ACLD). In 12 cases, the meniscus was also affected; in 9 cases, it was not. Detailed information is available in the participant log file and summarized in Table 1.

**Table 1:** Participant information for the ACL group and control group.

	ACL Group (n = 21; females: 11)	Control Group (n = 22; females: 12)	p-Value*
Age (years)	24.2 ± 3.5	26.5 ± 3.2	0.008*
Height (m)	1.8 ± 0.1	1.7 ± 0.1	0.568
Weight (kg)	69.6 ± 10.7	68.0 ± 14.8	0.689
BMI (kg/m^2^)	22.6 ± 1.7	22.4 ± 2.5	0.350
ACLR	19	n/a	
ACLD	2		
Leg dominance^a^	Right: 19	Right: 20	
	Left:2	Left: 2	
Injury side	Dominant leg: 9	n/a	
	Non-dominant leg: 12		
Time since injury (year)	3.9 ± 2.5	n/a	
Reinjury on ipsilateral side	0/21	n/a	
Graft type	BPTB: 4	n/a	
	ST: 11		
	QT: 4		
Meniscus affected	Yes: 12	n/a	
	No: 9		
Physical activity (day/week)	4.9 ± 1.3	4.6 ± 1.1	0.399
Physical activity (minutes/session)	89.3 ± 34.0	94.1 ± 31.5	0.580
IKDC (%)	93.2 ± 7.8	n/a	
ACL-RSI scale	67.0 ± 17.9	n/a	
Tampa scale of kinesiophobia (score)	21.3 ± 4.8	n/a	
Jump height (cm)	Pre-fatigue: 11.7 ± 4.3	Pre-fatigue: 12.3 ± 3.2	Main effect fatigue: 0.001
	Post-fatigue: 8.9 ± 3.8	Post-fatigue: 9.6 ± 3.1	
BORG CR10 scale (score)	Pre-fatigue: 0.6 ± 0.6	Pre-fatigue: 1.2 ± 1.5	Main effect fatigue: 0.001
	Post-fatigue: 6.0 ± 1.0	Post-fatigue: 6.7 ± 1.5	

Values are represented as mean ± standard deviation for continuous data and as frequencies for nominal data.

^a^Leg dominance was defined as the preferred kicking leg. ACLR: Anterior cruciate ligament reconstruction, ACLD: Anterior cruciate ligament deficiency. ST: semitendinosus-gracilis tendon graft, BPTB: bone–patellar tendon–bone graft, QT: quadricep tendon graft. IKDC: International Knee Documentation Committee. ACL-RSI scale: Anterior Cruciate Ligament Return to Sport after Injury scale. *p < 0.05.

Inclusion criteria for both groups required: (1) no lower extremity injuries within the six months preceding the study, and (2) no history of severe ankle injuries. For the ACL group, additional inclusion criteria were: (1) a history of one or two ACL injuries on the same limb (ipsilateral), and (2) clearance to return to level I sports^31,32^ (multidirectional field sports characterized by jumping, pivoting, and cutting movements) for at least one year before participation.

All participants were students of the University of Innsbruck. Ethical approval for the study was obtained from the local ethics board of the University of Innsbruck (Certificate 98/2022). The study procedures adhered to the ethical principles outlined in the Declaration of Helsinki, and all participants provided written informed consent before testing.

### Experimental protocol

Anthropometric, ACL injury-related data (International Knee Documentation Committee^33^, Tampa scale of kinesiophobia^34,35^, ACL Return to Sport after Injury scale^36^), and physical activity-related data were collected first. Limb dominance was defined by the preferred kicking leg. Before a standardized warm-up, participants were given standardized footwear (Adidas Handball Spezial). A five-minute warm-up on a bike ergometer was conducted, followed by three submaximal bilateral and unilateral countermovement jumps (CMJ).

Before testing, the maximal vertical jump height was measured using a unilateral CMJ and the BORG CR10 scale^37^ was used to assess perceived exertion as an indicator of participants’ fatigue^38^. The Borg CR10 is a category-ratio scale ranging from 0 (no exertion) to 10 (maximal exertion). For the ACL group, this involved the leg that was previously injured, while for the control group, the leg was randomly selected. Participants were instructed to stand on a force plate with one leg, place their hands on their hips, and maintain their swing leg at 90° knee flexion. They were then required to perform a maximal vertical jump and land on the same foot. Using the swing leg for propulsion was not permitted.

**Fig. 1:**
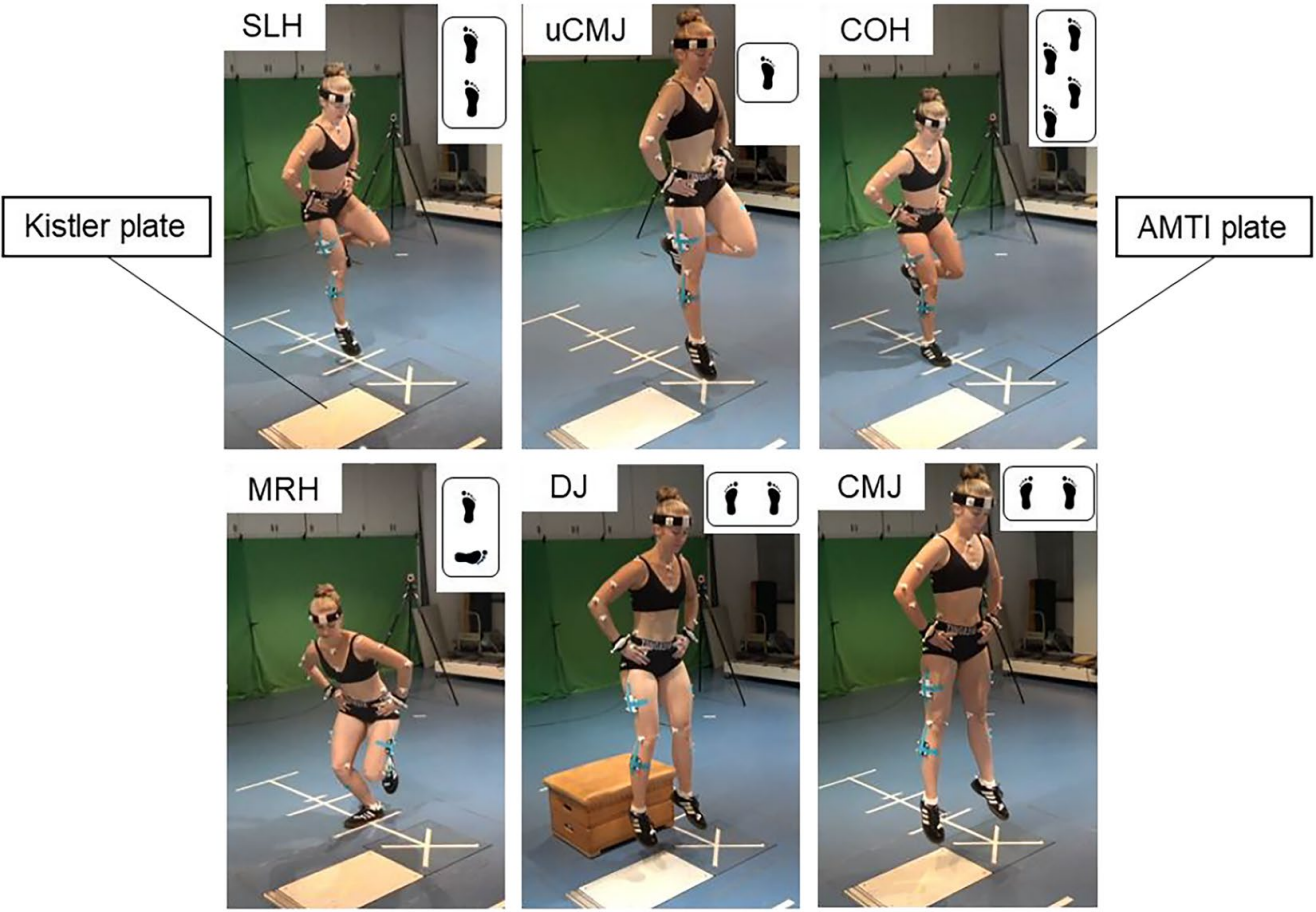
Jump-landings tasks. Upper panel from left to right: single-leg forward hop (SLH), single-leg countermovement jump (uCMJ), single-leg cross-over hop (COH); lower panels from left to right: single-leg 90° medial rotation hop (MRH), bilateral countermovement jump (CMJ), bilateral drop jump (DJ). White force plate represents the Kistler force plate and blue force plate represents the AMTI force plate. Note: The participant shown in the images provided written informed consent for the publication of their image.

For the jump protocol, participants performed six different jump-landing tasks before and after a fatiguing protocol. The following tests were performed: single-leg forward hop (SLH), single-leg countermovement jump (uCMJ), single-leg 90° medial rotation hop (MRH), single-leg crossover hop (COH), bilateral countermovement jump (CMJ), and a bilateral drop jump (DJ) (e.g, Fig. 1). For the SLH, participants started at a standardized distance of 100% of their leg length^10^ away from a force plate. Leg length was defined as the distance from the anterior superior iliac spine to the lateral malleolus of the dominant leg. They were instructed to stand on the leg to be tested, hop in a forward direction, and land on the same leg onto the middle of the force plate^39^. For the single-leg CMJ, participants stood with the leg to be tested on the middle of the force plate. The jumps started with a countermovement to a self-selected depth before accelerating in a vertical direction as fast and as high as possible^40^. They were additionally instructed to keep their swing leg in 90° knee flexion, and it was not allowed to use the swing leg for propulsion. For the COH, participants stood on the leg to be tested, and performed three consecutive hops in the forward direction, crossing over a center strip on each hop^39^. The distance between the landing marks was standardized at 50 cm for the specific reason that the jump had to be within the volume of our 3D motion capture device. For the MRH, participants were instructed to stand on the leg to be tested at a distance of 50% of their leg length away from a force plate, ensuring that the inner side of the foot is aligned perpendicular to the hopping direction^20^. The hop was executed in the transversal plane, involving a 90° rotation in the medial direction during the swing phase. Upon landing, the foot was to be oriented in the forward direction^20^. For the bilateral CMJ, participants stood with the left leg on one force plate and the right leg on another force plate. The jumps started with a countermovement to a self-selected depth before accelerating in a vertical direction as fast and as high as possible. The DJ was performed from a 30 cm box. Participants were instructed to step off the box, absorb the landing by making a downward movement, and then transition into an upward jump, aiming for maximum height^41^. For all jump landings, participants were required to keep their hands on their hips and keep the lowest knee flexion position after landing for at least two seconds. Further, they were instructed to stabilize the whole body while landing.

The jump-landings were considered successful if the participants landed in the middle of the force plates, did not lose their balance, did not remove their hands from their hips, and held the final landing position in the lowest knee flexion position for at least 2 seconds. Unsuccessful trials were repeated until three successful trials were obtained. Data were collected for both legs, and three trials per leg were performed for all jumps, except for the COH jump where only one trial per leg was performed. Three repetitions were performed for the bilateral CMJ and DJ. In total—accounting for both legs and both non-fatigued and fatigued conditions—12 trials were performed for the SLH, uCMJ, and MRH; 4 trials for the COH; and 6 trials for the bilateral CMJ and DJ. Before the jump protocol, a static trial was recorded to later scale the musculoskeletal model.

**Fig. 2:**
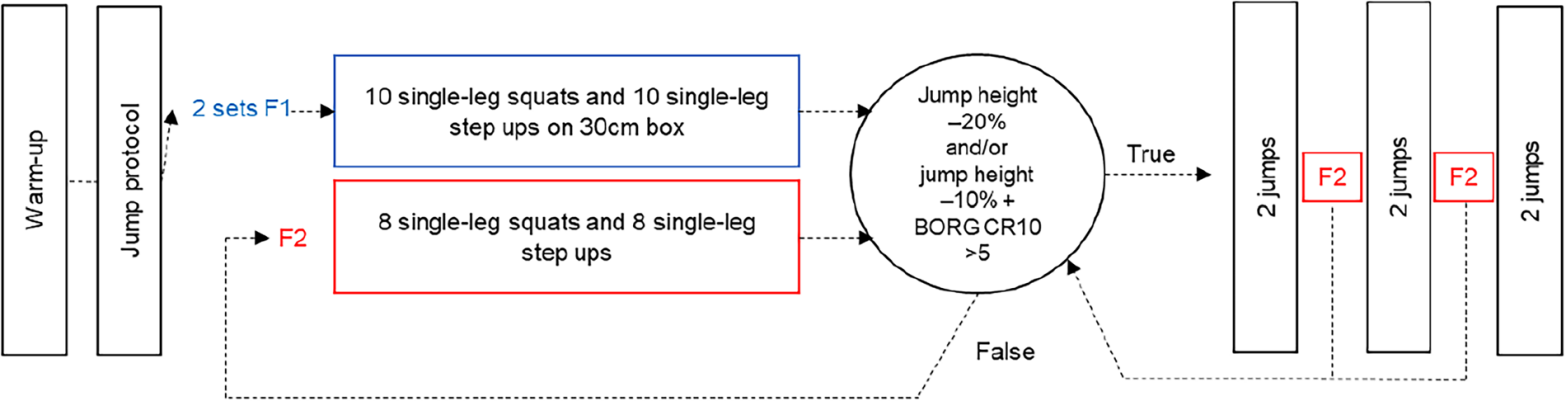
Schematic of the fatiguing protocol. F1: initial fatigue protocol to achieve fatigue; F2: second fatigue protocol executed if fatigue criteria (black circle) were not met, and executed after 2 jumps to ensure that the criteria were still met.

Following the completion of the jumps in the non-fatigued state, a peripheral fatigue protocol was initiated. Participants performed two different sets (F1 and F2, e.g. Fig. 2) of single-leg squats and single-leg step-ups. For the single-leg squats, participants lowered their bodies until their knees were at a 90° angle, lightly touching a 30 cm box with their bottom. The step-ups were done on a 30 cm box. In set F1, participants completed two sets of 10 repetitions for each exercise. In F2, they performed 8 repetitions for each exercise. After finishing the fatigue sets, the maximal jumping height was measured. Fatigue was defined by either 1) a 20% reduction in maximal jump height or 2) a 10% reduction in maximal jump height along with a rating on the BORG CR10 Scale^37^ higher than 5. If participants met either of these criteria, they immediately proceeded with two randomly selected jump-landing tasks from the jump protocol. If none of the criteria were met, F2 was repeated until one of the criteria was fulfilled (e.g. Fig. 2). To maintain the level of fatigue, F2 was repeated after performing two jump-landings tasks. Maximal jump height was calculated using the impulse-momentum theorem by a custom-written LabVIEW program^42^. The mean of three jumps was calculated to then define the 20% and 10% fatigue criteria.

### Instrumentation

Whole-body kinematics and ground reaction force data were recorded using a 10-camera motion tracking system (250 Hz; 8 Bonita camera and 2 Vero v2.2 cameras; Vicon Motion Systems Ltd., Oxford, UK) and two force plates (1000 Hz; AMTI, Watertown, Massachusetts, MA, USA; Kistler, Winterthur, Switzerland). Time-synchronized camera and force plate data were recorded in Nexus software v.2.14.0 (Vicon Motion Systems Ltd., Oxford, UK).

For body movement tracking, 59 retro-reflective markers were placed on the participants´ skin and shoes. Thereto, the Vicon Plug-in Gait full-body model was supplemented with additional markers: left and right medial ankle and knee joint, greater trochanter, crista iliaca, and cluster markers on the thigh and shank (e.g. Fig. 4). A detailed marker description can be found in the dataset^43^ in the Supplementary Material folder (e.g., folder “data processing” – subfolder “supplementary Material” - Table 1).

### Data processing

#### Initial data processing

**Fig. 3:**
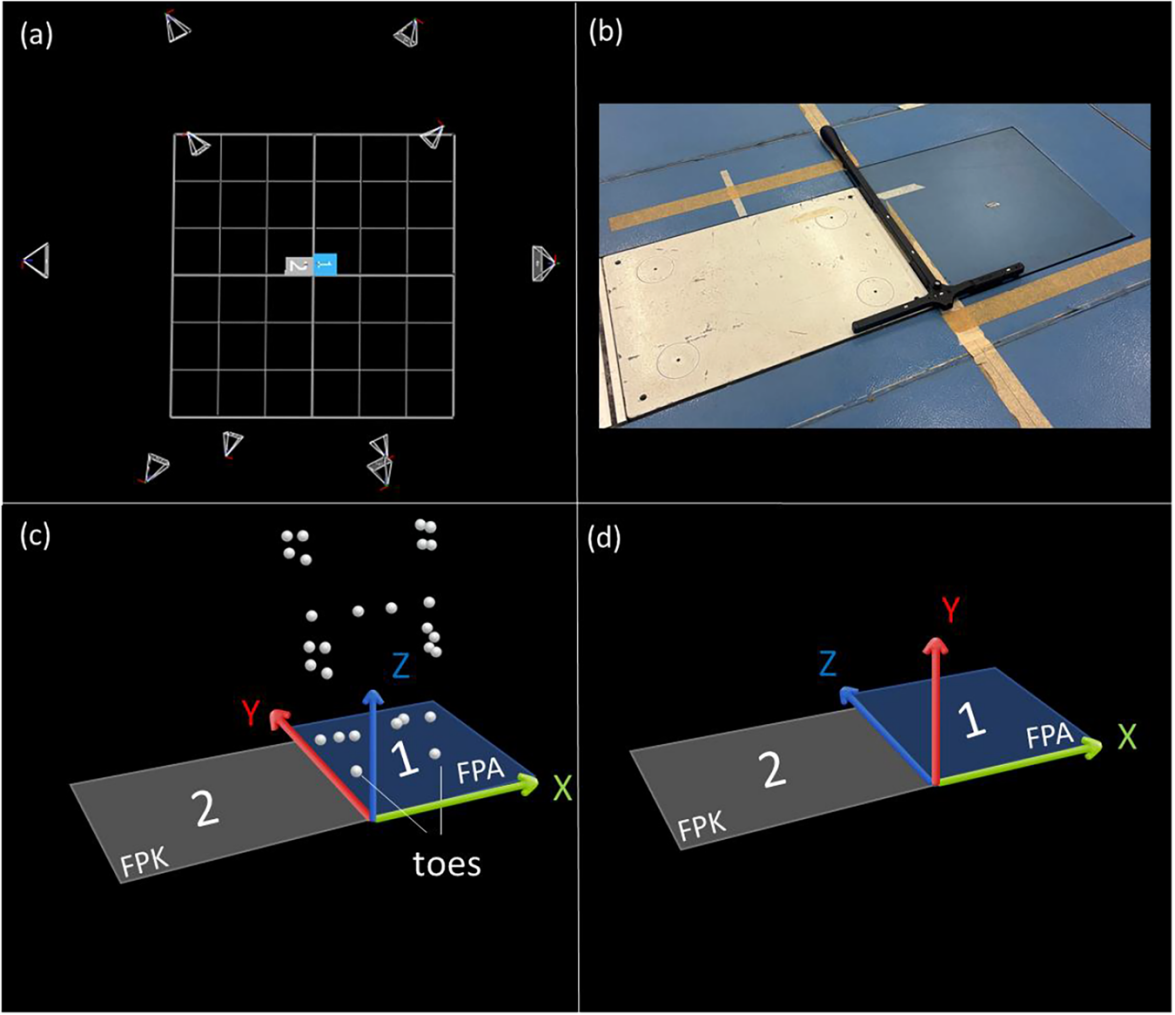
Motion capture environment with the 10-camera setup (**a**) top view, (**b**) placement of the LED active wand used to define the origin, (**c**) origin and orientation of the axes defining the reference coordinate system for the marker tracking system and force plates in the .c3d files, (**d**) rotated coordinate system used for further processing in OpenSim. FPK = Force plate Kistler (2); FPA = Force plate AMTI (1).

Marker trajectories were first reconstructed, labeled, gap-filled, and exported with the force plate data as .c3d files using Vicon Nexus software (version 2.14.0, Vicon, Oxford, UK). The data was then rotated to align with OpenSim’s coordinate system (e.g. Fig. 3(d)). Marker trajectories were filtered using a third-order, zero-lag, low-pass Butterworth filter with a 15 Hz cut-off frequency^44^ and then written to.trc files to enable their subsequent processing in OpenSim 4.3 software.

**Fig. 4:**
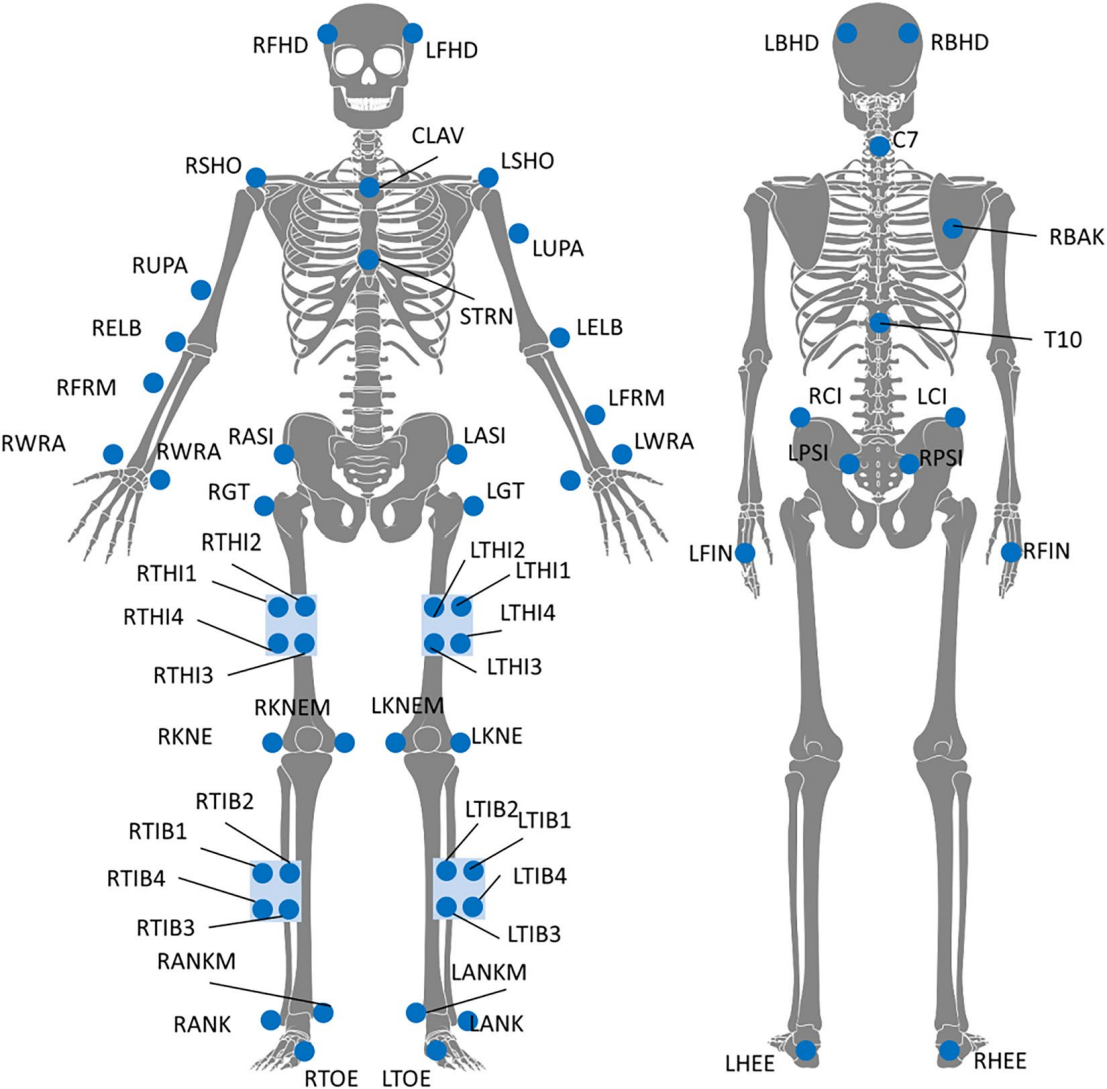
Markerset including 43 reflective markers and 4-marker clusters. The back view displays only those markers that are not visible in the front view and omits markers already shown from the front perspective.

Each subject-specific model was manually scaled in OpenSim using the static trials and the corresponding scaling setup file^45^. The model used was a modified version of the generic whole-body musculoskeletal model by Catelli and colleagues^46^. It was adapted to incorporate two additional degrees of freedom: knee abduction-adduction and knee internal-external rotation. Furthermore, the pronation-supination degree of freedom of the ankle joint and the metatarsophalangeal joint were locked. Inverse kinematics^45^ was then computed via the Matlab-OpenSim scripting interface and the resulting joint angles for all movements were stored in a Matlab structure (The MathWorks, Inc., Version R2021a, Natick, MA, USA). Subsequently, the OpenSim Analyze Tool was used to extract center of mass (COM) position, velocity, and acceleration data, which was stored in the same Matlab structure.

The vertical ground reaction force (GRF) was filtered using a third-order, zero-lag, low-pass Butterworth filter with a 15 Hz cut-off frequency based on a previous study^44^ and resampled from 1000 Hz to match the trajectory data rate of 250 Hz. To support potential inverse dynamics analyses, the same cut-off frequency was used for both GRF and marker trajectory data, in line with recommendations in the literature^47^. Initial contact events were then identified as instances where the resampled and filtered vertical GRF exceeded 20 N^48^. For the drop jump (DJ) trials, both the first and second ground contact events were calculated and stored in the corresponding Matlab structure.

The Matlab structure includes initial contact events, COM data, joint angles, and filtered rotated marker trajectories. All raw data, including unfiltered trajectories, ground reaction forces, and center of pressure data, can be extracted from the original .c3d files.

For the demographic data (e.g. Table 1), the mean and standard deviation were calculated for age, height, weight, BMI, time since injury, physical activity days per week, and minutes per session for each group. Further jump height and BORG CR10 scores before and after the fatiguing protocol are shown in Table 1. The normality of these variables using the Shapiro-Wilk test was assessed, followed by independent t-tests to determine whether there were significant differences between the groups. For the jump height, a repeated measures ANOVA with the between-subject factor ‘group’ was calculated to determine if fatigue had a significant effect on jumping height.

## Data Records

**Fig. 5:**
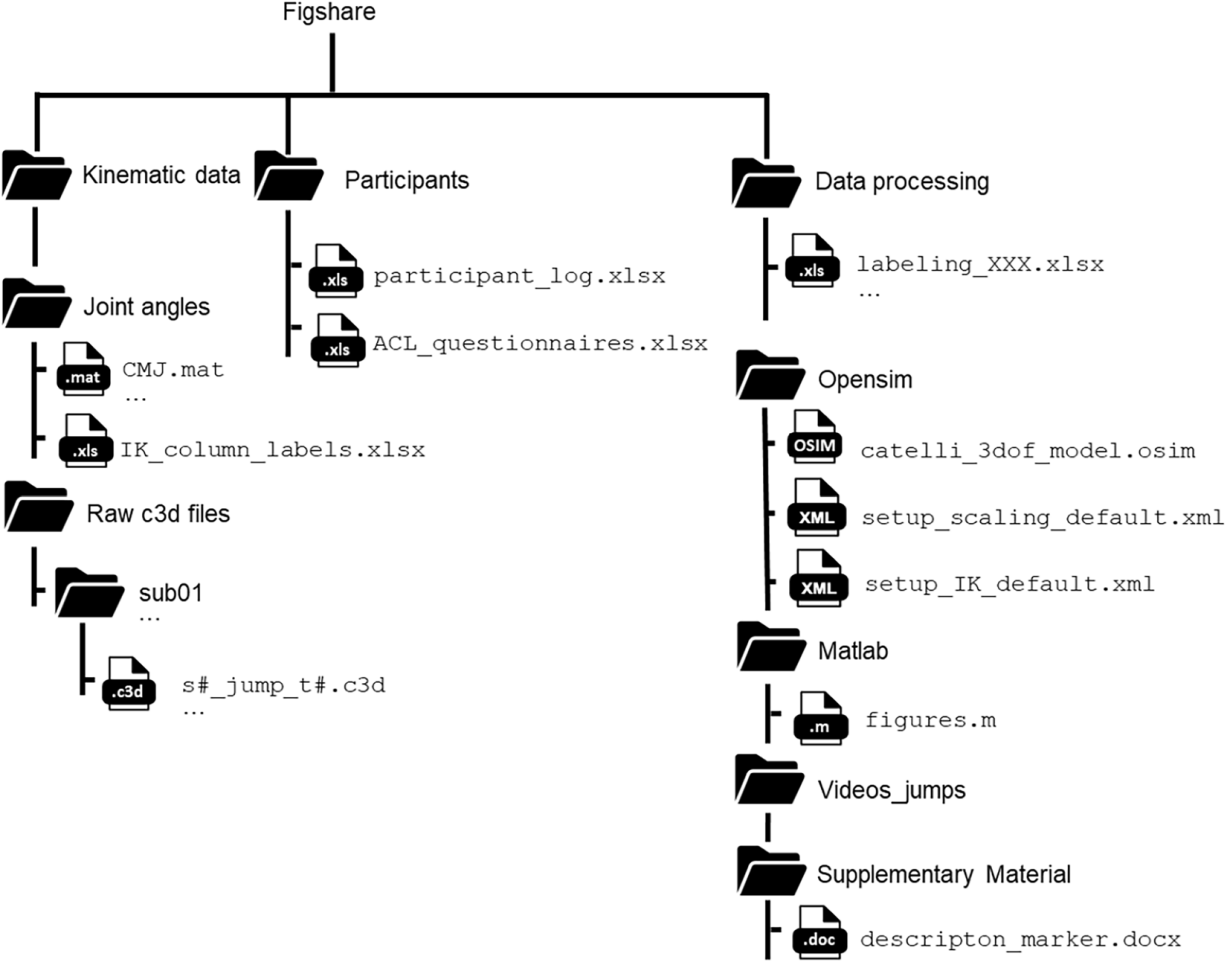
Folder structure of the dataset and file names.

The dataset is available on Figshare (CC BY 4.0)^43^ and is organized into three main folders: *participants*, *kinematic data*, and *data processing* (e.g. Fig. 5).

The *participants* folder contains the file participant_log.xlsx, which includes detailed demographic data (group, gender, age, height, weight, BMI, leg length of the dominant leg), sports-related data (physical activity (days/week), duration of a session (minutes), main sport), ACL injury-related data (injured leg, course of injury, reconstruction, graft type, meniscus affected, year of the injury, time since the injury, level of activity regained, rehabilitation duration in month, duration of one rehabilitation session). The file also includes results from vertical jump height measurements, as well as the BORG CR10 scores.

Additionally, ACL injury-related data collected from three questionnaires (IKDC, Tampa scale, ACL-RSI) are provided in the Excel file titled ACL_questionnaires.xlsx.

An overview of the participants’ information, calculated as means and standard deviations, can be found in Table 1.

In the *kinematic data* folder, the folders *joint_angles* and *raw_c3d_files* can be found.

The *raw_c3d_files* folder contains subfolders for each participant, including raw data from marker trajectories and force plate measurements (GRF and center of pressure data) for all jump-landing tasks and the static trial in .c3d files.

**Fig. 6:**
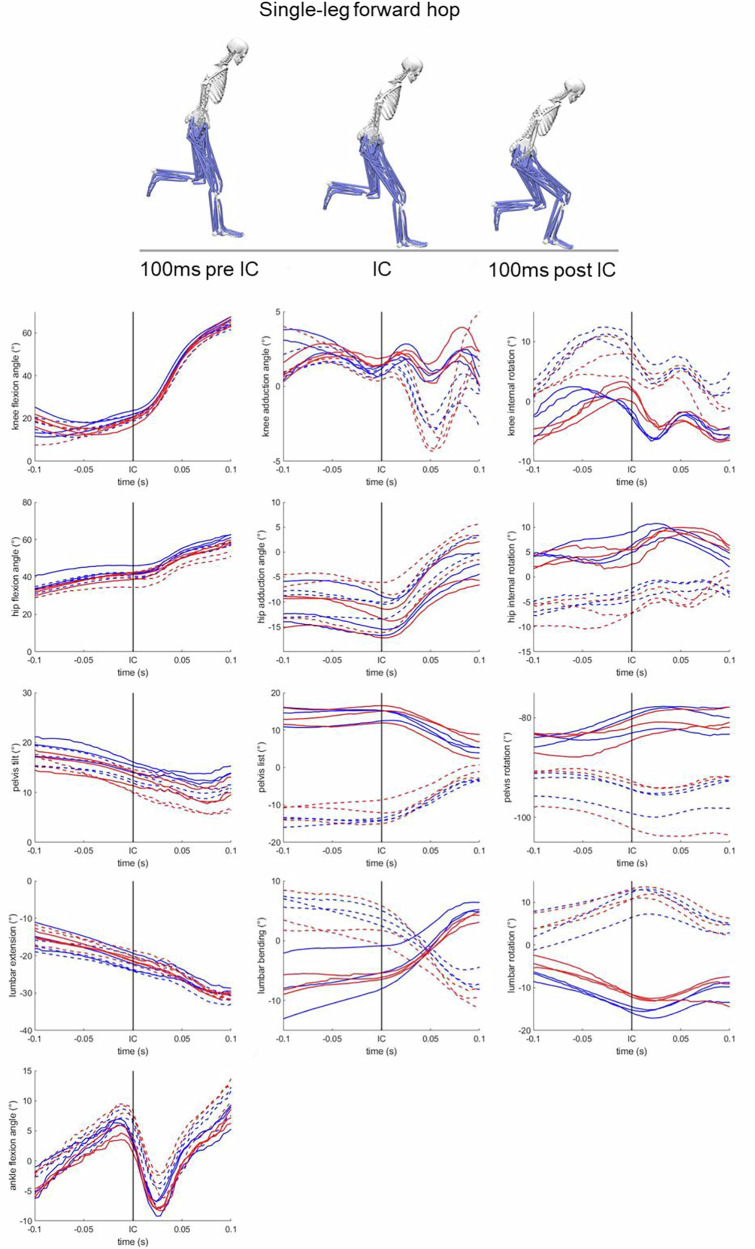
An example of joint angle waveforms (sub 1, SLH) ranging from −100 ms to +100 ms before and after initial contact (IC, vertical black line) of the landing. The 13 joint angles include knee and hip flexion/extension, knee and hip abduction/adduction, knee and hip internal/external rotation, trunk flexion/extension, trunk bending, and trunk rotation, pelvis tilt, pelvis list, pelvis rotation, and ankle flexion/extension angle. Solid blue lines represent right leg trials in the non-fatigued state, while solid red lines represent right leg trials in fatigued state. Dashed blue lines indicate left leg trials in non-fatigued, and dashed red lines the left leg trials in fatigued state.

The *joint angles* folder contains Matlab files for all six jump-landing tasks (e.g. CMJ.mat) and the file IK_column_labels.xlsx., which describes all joint angle labels in detail. Each.mat file includes cell arrays and structures containing the following fields: initial contact events, center of mass data, joint angle waveforms, and filtered marker trajectories. These are organized across trials, participants, conditions (non-fatigued and fatigued), and limbs. For illustration purposes, thirteen joint angles relevant to ACL injury-related research were extracted and are presented in Fig. 6. These include knee and hip flexion/extension, abduction/adduction, and internal/external rotation, ankle flexion/extension, trunk flexion/extension, lateral bending, and rotation as well as pelvis tilt, list, and rotation. As shown in the Fig. 6, some angles show mirrored patterns, e.g., lumbar bending or rotation. This should be taken into account when comparing the right and left legs.

The *data processing* folder includes the files labeling_jump_side.xlsx, as well as two folders: *Matlab* and *OpenSim*. The Excel f*iles* contain metadata for each trial, including jump type, subject ID, group, gender, leg side, condition (non-fatigued or fatigued), leg dominance, fatigued leg, ACL-injured leg. They also include columns labeling trials that were missing or excluded during subsequent processing steps, as well as a column indicating the reason for missing or excluded trials and whether a change in jump direction orientation occurred (e.g., COH jump-landing task).

The *Matlab* folder contains the script figures.m, which can be used to reconstruct and visualize the joint angle waveforms presented in Fig. 6,serving as an example of how to access the data.

The *OpenSim* folder includes all relevant setup files used for post-processing the raw data in the OpenSim environment. This includes the modified musculoskeletal model^46^ (catelli_3dof_model.osim), the scaling setup file (setup_scaling_default.xml), and the inverse kinematics setup file (setup_IK_default.xml).

The *Videos_jumps folder* provides video files (e.g., CMJ.webm) that visualize the OpenSim model performing each of the six different jump tasks.

Lastly, the *Supplementary_Material* folder contains a document (description_markers.docx) that includes a table defining the marker acronyms along with their corresponding anatomical landmarks.

### Missing data

With 43 participants and 52 planned jumping trials per participant, the dataset^43^ could include up to 2236 trials. However, 35 trials were not recorded due to issues such as participant discomfort or marker loss (e.g., from sweat), reducing the available raw data to 2201 trials. These missing trials have no corresponding .c3d files and are labeled with a “1” in the file labeling_jump_side.xlsx, with the reason noted in the “notes” column. Additionally, two trials, although recorded, were excluded from joint angle calculations due to abnormal motion patterns. These trials are still available as raw .c3d files but were not included in the processed joint angle dataset, resulting in 2199 trials with complete joint angle data (e.g. Table 2).

**Table 2:** Overview of recorded and missing trials for raw data and processed joint angle data.

Data	Total	Missing	Note
Raw .c3d	2201	35	Trials not recorded and therefore not present in the dataset; marked as “1” in labeling_jump_side.xlsx.
			**sub32**: DJ_f_t1-3
			**sub39:** SLH_r_t2; MRH_f_r_t3
			**sub40:** SLH_f_r_t1-3;
			CMJ_f_r_t1-3; MRH_f_r_t1-3; COH_f_r_t1
			**sub44**: SLH_r_t3; SLH_l_t3; SLH_l_t3; CMJ_r_t3; CMJ_l_t3; CMJ_f_r_t3; CMJ_f_l_t3; MRH_l_t3; MRH_r_t3; MRH_f_l_t3; MRH_f_r_t3; CMJ_t3, CMJ_f_t3; DJ_t1-3; DJ_f_t1-t3.
joint angles Jump.mat	2199	35 + 2	2 trials excluded from processing due to abnormal motion; raw .c3d files still available.
			**sub29:** CMJ_r_t2
			**sub39:** MRH_f_l_t3

The table shows the total number of trials, number of missing trials, and specific notes indicating participant IDs and tasks for which data are missing.

### System setup

The dataset^43^ was collected under controlled laboratory conditions. The 3D motion capture volume was powered on for at least 30 minutes prior to calibration. Calibration, including camera calibration and volume origin setting, was performed before each session according to the manufacturer’s instructions using an LED active wand. The origin of the coordinate system was positioned between the two force plates. The active wand was placed in such a way that its “stem” (long part of the T) was aligned along the y-axis, while the “crossbar” (short part of the T) was oriented parallel to the x-axis (e.g. Fig. 3(b)). Time-synchronized camera and force plate data were recorded using Nexus software, with all devices integrated into the Vicon system network to ensure synchronized data acquisition. The force plates were zeroed in Vicon Nexus before each trial. Marker placement was performed by the same researcher for all participants. Participant, sport-related, and injury-related information was obtained via self-reported questionnaire, except for weight and height, which were measured by the researcher at the beginning of each session.

## Technical Validation

First, joint angle time-series were computed for all jump-landing tasks using OpenSim, and representative joint angle waveforms for thirteen kinematic variables were plotted using the included Matlab script (e.g., Fig. 6). These visualizations show movement patterns for knee, hip, ankle, pelvis, and trunk segments under both fatigued and non-fatigued conditions. Second, the dataset^43^ has been used in a related peer-reviewed study^30^, in which machine learning models successfully differentiated between ACL-injured and control participants, showing that the data support predictive classification based on biomechanical features. Third, the comprehensive metadata (e.g., jump type, leg side, fatigue status, injury side, and leg dominance) allows users to conduct subgroup and symmetry analyses or explore the effect of an ipsilateral injury to the contralateral limb. Finally, the availability of both marker trajectories and synchronized force plate data allows for advanced biomechanical analyses, including joint moment calculations.

## Usage Notes

The .c3d files are named according to a specific scheme and always include the participant ID, the name of the jump, whether it was a fatigued trial, the tested leg, and the trial number. For non-fatigued trials, the naming convention is as follows: s01_CMJ_r_t1, while for fatigued trials, it is s01_CMJ_f_r_t1. A full list of the acronyms used in the filenames, along with their meanings, is provided in Table 3.

**Table 3:** Acronyms used in file naming.

Acronym	Meaning	Description
s01	Subject 01	Participant identifier (Subject 1)
uCMJ	Unilateral countermovement Jump	Type of jump performed
SLH	Single-leg forward hop	
COH	Single-leg cross-over hop	
MRH	Single-leg 90° medial rotation hop	
CMJ	Bilateral countermovement jump	
DJ	Bilateral drop jump	
f	Fatigued	Trial was performed under fatigued condition
r/l	Right leg/left leg	Right leg or left leg was the tested limb
t1	Trial 1	First repetition of this jump condition

Each filename encodes key metadata, including subject ID, jump type, fatigue condition, tested leg, and trial number.

The .c3d files can be read and visualized in the Motion Kinematic and Kinetic Analyzer (Mokka; http://biomechanical-toolkit.github.io/mokka/index.html). The.mat files can be read and analyzed in the Matlab software (The Mathworks, Inc., Natick, MA, USA).

The data can be utilized in two ways: either by directly using the processed marker trajectories, joint angle and center of mass data based on the timing of the initial contact as provided in the Matlab files or through custom-written scripts analyzing the contents of the raw .c3d files using additional/other biomechanical computations than described here.

## Data Availability

The codes for the figures of the joint angles can be found in Figshare. For the Matlab-OpenSim scripting interface, the provided instructions and codes from OpenSim were used (Scripting with Matlab - OpenSim Documentation - OpenSim).
